# Characterization of T-Bet and Eomes in Peripheral Human Immune Cells

**DOI:** 10.3389/fimmu.2014.00217

**Published:** 2014-05-14

**Authors:** James J. Knox, Gabriela L. Cosma, Michael R. Betts, Laura M. McLane

**Affiliations:** ^1^Department of Microbiology, Perelman Institute for Immunology, University of Pennsylvania, Philadelphia, PA, USA; ^2^Department of Immunology, Thomas Jefferson University, Philadelphia, PA, USA

**Keywords:** T-box transcription factors, T-cells, NK cells, B-cells

## Abstract

The T-box transcription factors T-bet and Eomesodermin (Eomes) have been well defined as key drivers of immune cell development and cytolytic function. While the majority of studies have defined the roles of these factors in the context of murine T-cells, recent results have revealed that T-bet, and possibly Eomes, are expressed in other immune cell subsets. To date, the expression patterns of these factors in subsets of human peripheral blood mononuclear cells beyond T-cells remain relatively uncharacterized. In this study, we used multiparametric flow cytometry to characterize T-bet and Eomes expression in major human blood cell subsets, including total CD4^+^ and CD8^+^ T-cells, γδ T-cells, invariant NKT cells, natural killer cells, B-cells, and dendritic cells. Our studies identified novel cell subsets that express T-bet and Eomes and raise implications for their possible functions in the context of other human immune cell subsets besides their well-known roles in T-cells.

## Introduction

The transcription factors T-bet (T-box expressed in T-cells) and Eomesodermin (Eomes) belong to the phylogenetically related family of T-box transcription factors that share a sequence-specific T-box DNA-binding domain first identified in the murine *Brachyury* gene (1). While members of this family are known to play diverse roles in various developmental processes (2, 3), the functions of T-bet and Eomes have been best described in the context of the mouse immune system.

T-bet was originally defined as the master regulatory transcription factor involved in promoting T_H_1 CD4^+^ T-cell development while specifically inhibiting T_H_2 and T_H_17 lineage-defining programs in murine models (4–7). T-bet is known to modulate a number of genes involved in T-cell mobilization (CXCR3), cell signaling (IL12Rβ1), and cytolytic signaling molecules (IFNγ) (8). Additionally, high levels of T-bet expression are closely associated with cytotoxic CD8^+^ T-cell effector differentiation and function, including the upregulation of perforin and granzyme B in antigen-specific cells (9–12). T-bet has been implicated in sustaining memory subsets (13–16), however, T-bet levels decline as cells become more memory-like (17).

Eomesodermin was originally identified in *Xenopus* (18), and has since been found in many other vertebrates, where it plays key roles in mesoderm formation and early gastrulation events (18, 19). In the immune system, like T-bet, Eomes can positively influence the expression of IFNγ in CD8^+^ T-cells (13, 20, 21). In contrast to T-bet, Eomes expression increases as cells become more memory-like (10, 14, 16, 17) and Eomes knockout mice are deficient in long-term memory formation and fail to undergo homeostatic renewal (14, 16, 22) highlighting its critical role for memory differentiation.

Recently, evidence has emerged in mice that T-bet and Eomes may function in the context of other cells of the immune system; however, few studies have described the expression of these factors in human non-thymocyte immune cells. Additionally, few studies have investigated the co-expression of these factors within different immune cell subsets. In this study, we sought to broadly characterize the resting expression patterns of T-bet and Eomes in the context of a number of immune cells from normal human donors and to provide direct comparative data with identical optimal experimental conditions and cell sources to serve as a reference for future studies on these transcription factors in human lymphocytes. Using multiparametric flow cytometry, our results reveal some parallels between human and mouse models, however, we find key differences in specific cell subsets suggesting the role of these factors might not be identical in mouse and humans. Taken together, these studies suggest roles for these factors, both independently and together, beyond their known functions in CD4^+^ and CD8^+^ T-cells.

## Materials and Methods

### Human cells

Donor peripheral blood mononuclear cells (PBMCs) were collected after written, informed consent from the University of Pennsylvania’s Center for AIDS Research Human Immunology Core (IRB #705906) in compliance with IRB guidelines. PBMCs were cryopreserved in fetal bovine serum (FBS; Hyclone) containing 10% dimethyl sulfoxide (DMSO; Fisher Scientific) and stored at −140°C until further use.

### Flow cytometry analysis

Flow cytometry analysis was performed as previously described (10) using PBMCs from at least eight normal donors. Where appropriate, statistical analyses were performed using GraphPad Prism software (Version 5.0a). For these studies, non-parametric Wilcoxon matched paired *t* tests were used where Gaussian distribution is not assumed because we analyzed <25 subjects.

To identify CD4^+^, CD8^+^, and T-regulatory (T_reg_) T-cells, the following antibodies were used: α–CD3-BV570 (Biolegend), α–CD4-PE Cy5.5 (Invitrogen), α–CD8-BV605 (Biolegend), α–CD14/α–CD16/α–CD19-APC Cy7 (BD Bioscience), α–CCR7-BV711 (Biolegend), α–CD45RO-PE Texas Red (Beckman Coulter), α–CD27-FITC (eBioscience), α–CD25-PE Cy5 (Invitrogen), α–CD127-PE Cy7 (eBioscience), α–T-bet-PE (eBioscience), α–Eomes-Alexa647 (eBioscience), and α–Foxp3 Alexa700 (eBioscience).

To identify natural killer (NK), invariant natural killer (iNKT), and γδ T-cells, the following antibodies were used: α–CD3-BV570 (Biolegend), α–CD4-PE Cy5.5 (Invitrogen), α–CD8-BV605 (Biolegend), α–CD16-PE Cy5 (Biolegend), α–CD56-Alexa700 (Biolegend), α–CD19-BV785 (Biolegend), α–CD45RO-PE Texas Red (Beckman Coulter), α–γδ TCR-FITC (Biolegend), α–Vα24Jα18-PE Cy7 (Biolegend), α–T-bet-PE (eBioscience), and α–Eomes-eF660 (eBioscience).

To identify B-cell subsets, the following antibodies were used: α–CD3-BV570 (Biolegend), α–CD14/α–CD16-APC Cy7 (BD Bioscience), α–CD19-BV785 (Biolegend), α–CD20-PE Texas Red (BD Bioscience), α–IgD-Alexa700 (BD Bioscience), α–IgM-BV605 (Biolegend), α–CD38-BV421 (Biolegend), α–CD10-PE Cy5 (BD Bioscience), α–CD21-PE Cy7 (Biolegend), α–CD27-BV650 (Biolegend), α–IgG1-Alexa488 (Invitrogen), α–T-bet-PE (eBioscience), and α–Eomes-eF660 (eBioscience).

In addition to previously mentioned antibodies, the following were used to identify dendritic cell subsets: α–CD14-QD655 (Invitrogen), α–CD11c-FITC (BD Bioscience), α–HLADR-V450 (BC Bioscience), α–CD56-PE Texas Red (Invitrogen), and α–CD123 PE Cy5 (Biolegend).

## Results

### T-bet^hi^ and Eomes expression correlates with T_EM_ and effector CD8^+^ T-cells

T-bet and Eomes have been extensively studied in murine CD8^+^ T-cells and are critical for effector function and long-term memory formation, respectively (13, 20, 23–25). Recently, we described T-bet and/or Eomes in human CD8^+^ T-cell memory populations using various combinations of the memory markers CD27, CCR7, and CD45RO (9, 10, 26). While a fair amount is known about the relationship between T-bet and Eomes and the individual memory markers, the breakdown of their combined expression is not well defined.

**Figure 1:**
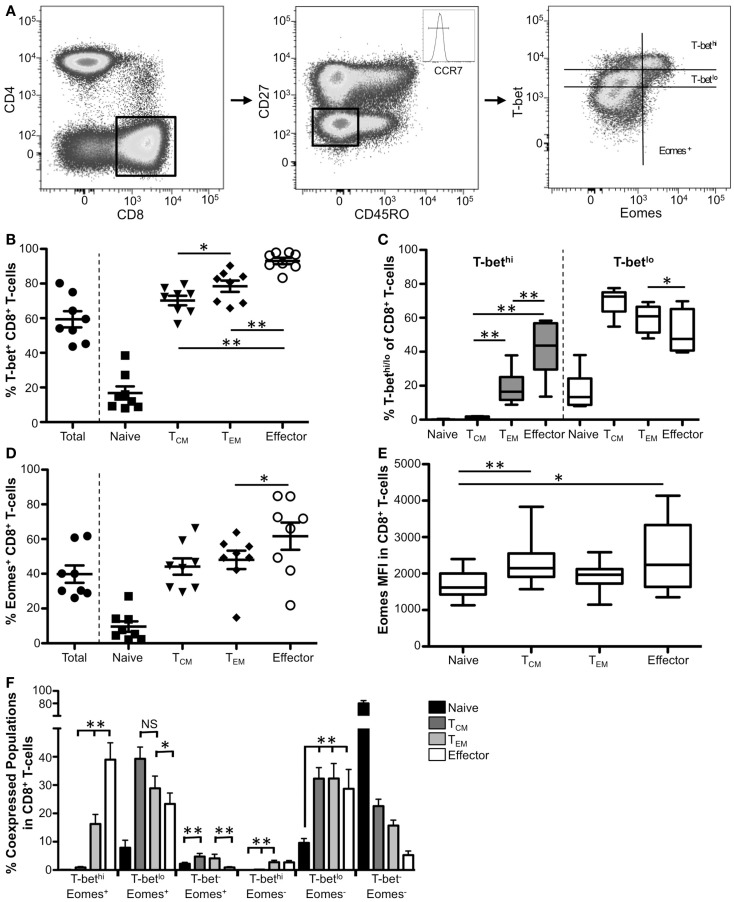
**T-bet and Eomes expression correlates with T_EM_ and effector CD8^+^ T-cells**. **(A)** Gating strategy for identifying CD8^+^ T-cell subsets. Flow cytometry data shown were gated as follows: singlets, lymphocytes, Aqua Blue^−^ (live cells), CD14^−^CD16^−^CD19^−^, CD3^+^, CD4^−^, CD8^+^. Boolean gating of CD27, CCR7, and CD45RO was used to define CD8^+^ subsets from eight normal donors. Effector CD8^+^ T-cells from a representative donor are shown. **(B)** The frequency of T-bet^+^ CD8^+^ T-cells within naïve (CCR7^+^CD45RO^−^CD27^+^), T_CM_ (CCR7^+^CD45RO^+^CD27^+^), T_EM_ (CCR7^−^CD45RO^+^CD27^−^), and effector (CD27^−^CD45RO^−^CCR7^−^) cells is shown. Each symbol represents an individual donor. **(C)** Graphical representation of the mean frequency of T-bet^hi^ (gray) and T-bet^lo^ (white) CD8^+^ T-cells is shown for each memory subset. The box and whisker graphs display 25–75% (box) and 10–90% (whisker). The line in the box represents the median value. **(D)** The frequency of CD8^+^ T-cells expressing Eomes is shown for each subset. **(E)** Median fluorescence intensity (MFI) is shown for Eomes within each subpopulation. **(F)** Co-expression of T-bet and Eomes within each memory subset is shown. *p < 0.04, **p < 0.004.

Based upon coordinate expression of CD27, CD45RO, and CCR7, we delineated peripheral blood CD8^+^ T-cells into six different populations, including naïve (CCR7^+^CD45RO^−^CD27^+^), central memory (T_CM_, CCR7^+^CD45RO^+^CD27^+^), transitional memory (CCR7^−^CD45RO^+^CD27^+^), effector memory (T_EM_, CCR7^−^CD45RO^+^CD27^−^), intermediate (CCR7^−^CD45RO^−^CD27^+^), and effector (CCR7^−^CD45RO^−^CD27^−^) CD8^+^ T-cells using a Boolean gating strategy (Figure 1A; Figure S1 in Supplementary Material). For simplicity, we focused on naïve, central memory, effector memory, and effector populations in Figure 1 (refer to Figures S1A–D in Supplementary Material for the more detailed breakdown of these populations). Representative gating for T-bet and Eomes expression within effector CD8^+^ T-cells from a normal human donor is shown in Figure 1A. As has been previously reported (10, 23, 26), T-bet has a graded expression pattern including distinct T-bet^hi^ and T-bet^lo^ populations (Figure 1A, right panel). Approximately 60% of total CD8 T-cells expressed T-bet (Figure 1B). While less than 20% of naïve CD8^+^ T-cells were T-bet^+^, significantly more T_CM_, T_EM_, and effector T-cells expressed T-bet (Figure 1B, data not shown). As shown previously (10, 26), the majority of T-bet^+^ cells within memory CD8^+^ T-cell populations were T-bet^lo^; however, as cells progress toward a more terminally differentiated phenotype, the frequency of T-bet^hi^ cells significantly increased (Figure 1C). Taken together, these data suggest that high levels of T-bet are likely crucial for function in effector and some effector memory CD8^+^ T-cells, whereas T-bet may play a less definitive role in naïve and T_CM_ differentiation and function.

We next investigated Eomes expression within memory CD8^+^ T-cell memory subsets. Less than half of total CD8^+^ T-cells expressed Eomes (Figure 1D). Like T-bet, Eomes is expressed most infrequently in naïve CD8^+^ T-cells. While the frequency of Eomes^+^ T_CM_, T_EM_, and effector CD8^+^ T-cells increased significantly compared to naïve T-cells (data not shown), we found no significant difference in the frequency of T_CM_ T-bet^+^ cells compared to T_EM_ or effector cells. Additionally, there is more Eomes per cell in memory CD8^+^ T-cells compared to naïve T-cells (when it is expressed) as measured by median fluorescence intensity (MFI) analysis (Figure 1E). Taken together, these data indicate that Eomes is expressed in the highest frequency in effector cells but, on average, these cells do not express more Eomes per cell compared to other CD8^+^ T-cell memory subsets.

Because T-bet and Eomes likely have both redundant and unique roles in CD8^+^ T-cells (20, 27), we next investigated the co-expression of these factors in the CD8^+^ T-cell memory populations (Figure 1F). Naïve cells were almost exclusively T-bet^−^Eomes^−^ (Figure 1F, black bars). T_CM_ CD8^+^ T-cells were mainly T-bet^lo^ Eomes^+/−^, or T-bet^−^ Eomes^−^ (dark gray bars). Like T_CM_, T_EM_ T-cells were mainly T-bet^lo^ Eomes^+/−^; however, T_EM_ had a significantly higher frequency of T-bet^hi^ Eomes^+^ compared to T_CM_. Further, effector CD8^+^ T-cells had the highest frequency of T-bet^hi^ Eomes^+^ CD8^+^ T-cells. Interestingly, T-bet^hi^ T_EM_ and effector CD8^+^ T-cells were almost exclusively Eomes^+^ suggesting high levels of T-bet correlate with Eomes expression and these factors could be cooperating to promote critical functions in these CD8^+^ T-cell subpopulations.

### T-bet and Eomes associate with T_EM_ and effector CD4^+^ T-cell populations

**Figure 2:**
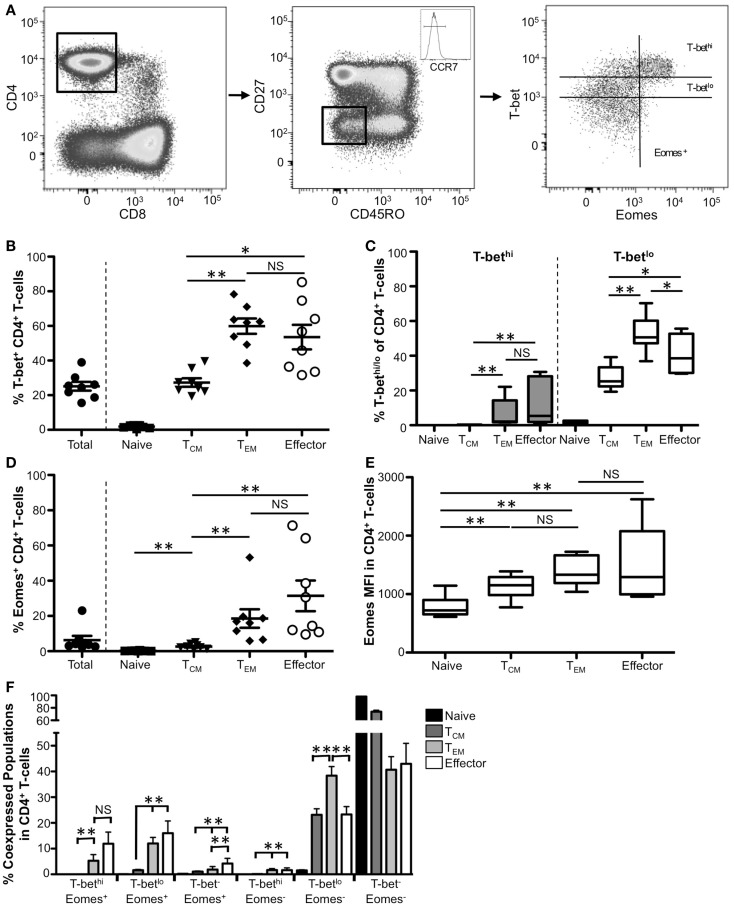
**T-bet and Eomes expression associates with T_EM_ and effector CD4^+^ T-cell populations**. **(A)** Gating strategy for identifying CD4^+^ T-cell subsets. Flow cytometry data shown were gated as follows: singlets, lymphocytes, Aqua Blue^−^ (live cells), CD14^−^CD16^−^CD19^−^, CD3^+^, CD8^−^, CD4^+^. Boolean gating of CD27, CCR7, and CD45RO was used to define CD4^+^ subsets from eight normal donors. Effector CD4^+^ T-cells from a representative donor are shown. **(B)** The frequency of T-bet^+^ CD4^+^ T-cells within naïve (CCR7^+^CD45RO^−^CD27^+^), T_CM_ (CCR7^+^CD45RO ^+^CD27^+^), T_EM_ (CCR7^−^CD45RO^+^CD27^−^), and effector (CD27^−^CD45RO^−^CCR7^−^) cells is shown. Each symbol represents an individual donor. **(C)** Graphical representation of the mean frequency of T-bet^hi^ (gray) and T-bet^lo^ (white) CD4^+^ T-cells is shown for each subset. The box and whisker graphs display 25–75% (box) and 10–90% (whisker). The line in the box represents the median value. **(D)** The frequency of CD4^+^ T-cells expressing Eomes is shown for each subset. **(E)** Median fluorescence intensity (MFI) is shown for Eomes within each subpopulation. **(F)** Co-expression of T-bet and Eomes within each memory subset is shown. *p < 0.04, **p < 0.004.

Initial work in mouse CD4^+^ T-cells first defined T-bet as the master regulator of T_H_1 development, with direct regulation of T_H_1-specific genes such as IFNγ (4). Early reports demonstrated expression of T-bet also within human CD4^+^ T-cells, but the patterns of T-bet expression within CD4^+^ T-cell memory subsets have remained unclear. We therefore sought to characterize T-bet expression in human CD4^+^ memory T-cell subsets. As with CD8^+^ T-cells, memory subpopulations were defined using the markers CD27, CD45RO, and CCR7 (Figure 2; Figures S1E–H in Supplementary Material). Representative flow plots for effector CD4^+^ T-cells are shown in Figure 2A. Overall, within our cohort, ~25% of total CD4^+^ T-cells expressed T-bet (Figure 2B). Few naïve human CD4^+^ T-cells were T-bet^+^, with a significant majority of T-bet expression found within the non-naïve CD4^+^ T-cell population (Figure 2B, data not shown). Approximately 20% of T_CM_ CD4^+^ T-cells expressed T-bet and that T-bet is almost exclusively T-bet^lo^ (Figures 2B,C). Significantly more T_EM_ and effector CD4^+^ T-cells expressed T-bet compared to T_CM_, however there was no difference between T_EM_ and effector CD4^+^ T-cells. While T_EM_ cells had the highest frequency of T-bet^lo^ cells, the frequency of T-bet^hi^ CD4^+^ T-cells increased significantly as cells progress toward a more effector-like phenotype suggesting that T-bet is likely more critical to the functions of CD4^+^ T_EM_ and effector cells than T_CM_ function.

Like T-bet, Eomes also plays a role in murine CD4^+^ T-cell differentiation. Eomes can induce both T_H_1 differentiation as well as expression of IFNγ and perforin in mouse CD4^+^ T-cells (13, 20, 21, 28). Eomes can also compensate for the loss of T-bet in CD4^+^ effector T-cells and drive polyfunctionality in human CD4^+^ T-cells (29); however, there are few *ex vivo* studies addressing Eomes expression in human CD4^+^ T-cells. In contrast to T-bet, very few human peripheral blood CD4^+^ T-cells express Eomes (Figure 2D). As cells become more effector-like, the frequency of Eomes^+^ cells significantly increases however the frequency only reaches ~25% in effector cells. Compared to naïve cells, we found a significantly higher Eomes MFI within each Eomes^+^ memory CD4^+^ T-cell subpopulation; however, while there was a trend toward higher Eomes MFI as cells become more effector-like, these differences were not significant (Figure 2E).

Because both T-bet and Eomes are known to induce IFNγ expression and multiple cytolytic functions in CD4^+^ T-cells (13, 20, 21, 30), we next examined co-expression of these factors within CD4^+^ T-cell subsets. We found significant increases in the frequency of all Eomes^+^ populations regardless of T-bet levels as cells progress to being more effector-like (Figure 2F). Like CD8^+^ T-cells, there were very few T-bet^hi^ cells that did not express Eomes, suggesting that high levels of these factors could cooperate to drive T_EM_ and effector differentiation and function; however, in contrast to murine studies, the majority of resting CD4^+^ T-cells were either T-bet^−^ Eomes^−^ or T-bet^lo^ Eomes^−^ suggesting that, at least in the context of resting peripheral blood CD4^+^ T-cells, T-bet, and Eomes may not contribute to resting CD4^+^ T-cell function.

### CD4^+^ T-regulatory cells express low levels of T-bet

**Figure 3:**
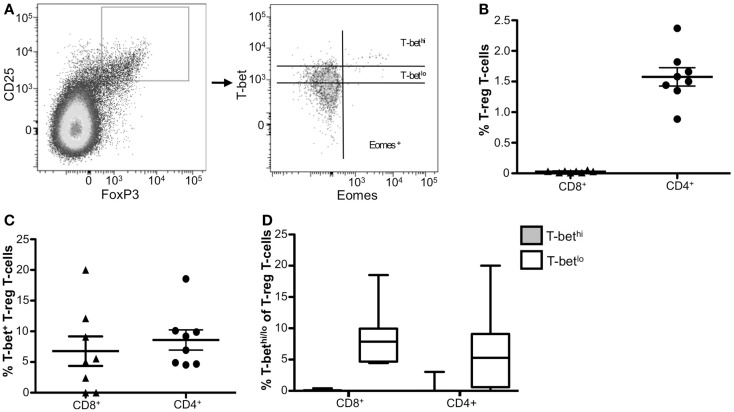
**Resting CD4^+^ T_reg_ cells express low levels of T-bet**. **(A)** Gating strategy for identifying CD4^+^ and CD8^+^ T_reg_ T-cells. Flow cytometry data shown were gated as follows: singlets, lymphocytes, Aqua Blue^−^ (live cells), CD14^−^CD16^−^CD19^−^, CD3^+^, CD8^+^ or CD4^+^, and CD25^+^ FoxP3^+^. A representative donor is shown. **(B)** The frequency of CD8^+^ and CD4^+^ T_reg_ T-cells is shown. **(C)** The frequency of T-bet^+^ CD8^+^ and CD4^+^ T_reg_ T-cells is shown. **(D)** Graphical representation of the mean frequency of T-bet^hi^ (gray) and T-bet^lo^ (white) CD8^+^ and CD4^+^ T_reg_ T-cells is shown. The box and whisker graphs display 25–75% (box) and 10–90% (whisker). The line in the box represents the median value. *p < 0.04, **p < 0.004.

T-regulatory cells function to suppress immune responses from other cell types to prevent hyperactivity or autoimmune disease. T_reg_ cells have been reported to upregulate T-bet *in vivo* during type-1 inflammatory responses (31); however, little is known about T-bet in circulating human T_reg_ cells. We next characterized T-bet and Eomes expression in resting CD8^+^ and CD4^+^ T_reg_ cells. We identified T_reg_ cells within both CD8^+^ and CD4^+^ populations using the markers CD25 and FoxP3 (Figure 3A). Only a small fraction of CD8^+^ T-cells were CD25^+^ Foxp3^+^, whereas CD4^+^ CD25^+^ Foxp3^+^ cells comprise about 1.5% of total PBMCs (Figure 3B). Within each of these T_reg_ populations, ~8% of cells expressed T-bet (Figure 3C) and this T-bet was almost exclusively T-bet^lo^ (Figure 3D). Eomes expression was not detected in circulating T_reg_ cells (data not shown). Taken together, these data indicate that neither T-bet nor Eomes likely contribute to resting human T_reg_ function.

### CD127 expression inversely correlates with T-bet expression in CD8^+^ T-cells

**Figure 4:**
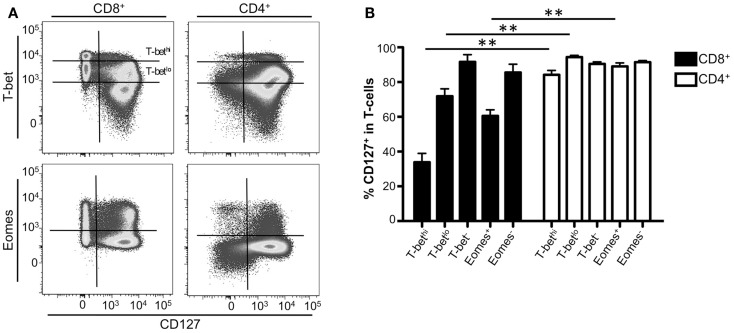
**CD127 expression inversely correlates with T-bet expression in CD8^+^ T-cells**. **(A)** Gating strategy for identifying CD8^+^ or CD4^+^ CD127^+^ T-cell memory subsets. Flow cytometry data shown were gated as follows: singlets, lymphocytes, Aqua Blue^−^ (live cells), CD14^−^CD16^−^CD19^−^, CD3^+^, CD8^+^ or CD4^+^, CD127^+^. A representative donor is shown. **(B)** The frequency of CD127^+^ CD8^+^ (black), or CD4^+^ (white) T-cells expressing T-bet or Eomes is shown. **p < 0.004.

We next investigated the relationship between long-term memory formation and expression of T-bet and Eomes in CD8^+^ and CD4^+^ T-cells through examination of IL-7 receptor (CD127) expression. T-cells are dependent upon IL-7 signaling for survival (32–36). In mice, naïve T-cells express CD127 and following T-cell receptor stimulation, CD127 is downregulated (32, 33, 37–39). Recent studies have suggested that the upregulation of T-bet in CD8^+^ T-cells results in the downregulation of CD127 (14, 23); however, a study in *Leishmania*-specific CD4^+^ T-cells suggest that the expression of T-bet did not inhibit CD127 expression, nor did the loss of T-bet result in upregulation of CD127 (40). Based on these results, we examined the relationship between T-bet and Eomes expression with CD127 in the context of human CD8^+^ and CD4^+^ T-cells. Correlating with previously published data (41, 42), CD127 was expressed in upwards of 85% of both CD8^+^ and CD4^+^ T-cells and the frequency of cells expressing CD127 decreased as cells became more effector-like (data not shown). Representative flow plots displaying the relationship between T-bet or Eomes and CD127 from one normal donor are shown in Figure 4A. In CD4^+^ T-cells, CD127 was expressed regardless of the presence or absence of T-bet or Eomes (Figure 4B, white bars). In contrast, as T-bet or Eomes expression increased in memory CD8^+^ T-cells, the frequency of CD127^+^ cells significantly decreased compared to CD4^+^ T-cells (Figure 4B, black bars). These results suggest that T-bet and Eomes may play a different role in controlling CD127 expression in CD8^+^ T-cells compared to CD4^+^ T-cells.

### T-bet and Eomes are co-expressed in human γδ T-cell subsets and CD4^−^CD8^−^ iNKT cells

**Figure 5:**
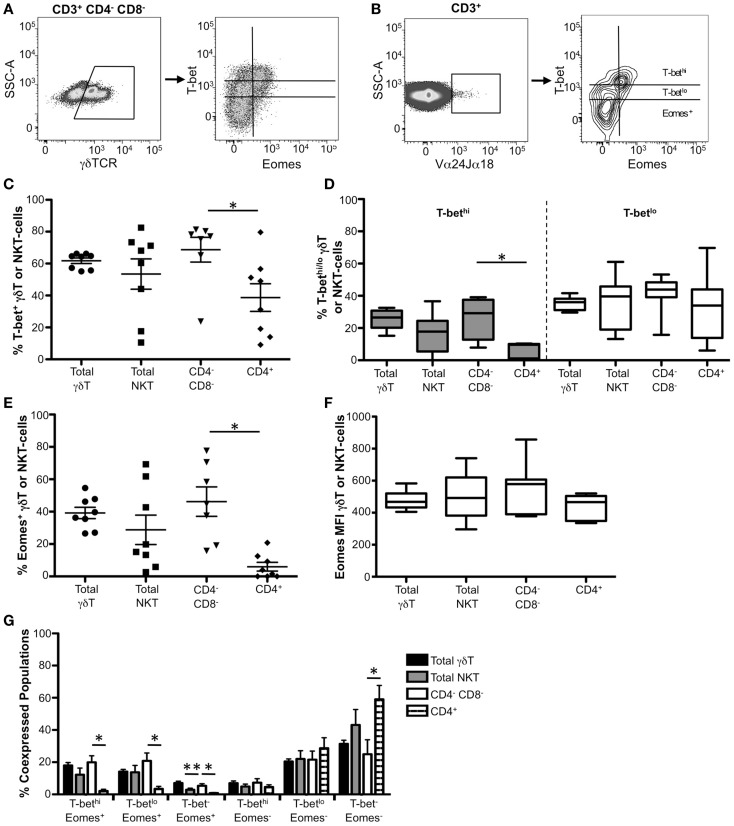
**Co-expression of T-bet and Eomes in γδ T-cells and CD4^−^CD8^−^ iNKT cells**. **(A,B)** Gating strategies for identifying **(A)** γδ T-cells and (B) iNKT cells. γδ T-cells and iNKT cells were gated as follows: singlets, lymphocytes, Aqua Blue^−^ (live cells), CD14^−^CD16^−^CD19^−^, CD3^+^, γδ TCR^+^ (γδ T-cells) or Vα24αJα18^+^ (iNKT cells). T-bet and Eomes expression within these cells from a representative donor is shown. **(C)** The frequency of T-bet expression in total γδ T-cells and iNKT cells is shown. Each symbol represents an individual donor. **(D)** Graphical representation of the mean frequency of T-bet^hi^ (gray) and T-bet^lo^ (white) expression in these populations is shown. The box and whisker graphs display 25–75% (box), 10–90% (whisker), and the median value (line). **(E)** The frequency of Eomes^+^ γδ and iNKT cells is shown for each cell subset. **(F)** Median fluorescence intensity (MFI) is shown for Eomes^+^ cells within each cell subset. **(G)** The frequency of T-bet and Eomes co-expression within each cell population is shown. *p < 0.04.

γδ T-cells and invariant natural killer T-cells (iNKT cells) are innate-like T-cell family members expressing T-cell receptors with restricted antigen recognition potential compared to classical αβ T-cells (43, 44). T-bet mRNA and protein, as well as Eomes mRNA, are detectable in mouse γδ T-cells, where it is suggested that they cooperate to control IFNγ production (45, 46). In mouse iNKT cells, T-bet is required for iNKT developmental progression and the acquisition of effector functions (21, 47). Expression of these transcription factors has not been comprehensively demonstrated in human γδ T and iNKT cells; therefore, we sought to characterize T-bet and Eomes expression in human γδ T and iNKT cells. We defined γδ T-cells as γδ TCR^+^ within CD3^+^CD4^−^CD8^−^PBMCs (Figure 5A); iNKT cells were identified within CD3^+^ PBMCs as TCR Vα24Jα18^+^ (Figure 5B). We further subdivided iNKT cells into CD4^−^CD8^−^ and CD4^+^ subgroups, the best described subpopulations of human iNKT cells (43). While we could detect CD4^−^CD8^+^ iNKT cells, they were infrequent and only detectable in 4/8 donors (data not shown).

As found in mice (21, 45–47), we observed T-bet expression in both resting γδ T and the major subsets of iNKT cells (Figure 5C). Approximately 60% of γδ T-cells and 50% of iNKT cells expressed T-bet, although we found considerable variation between donors within the iNKT population (Figures 5C,D, white bars). After subdividing iNKT cells into CD4^−^CD8^−^ and CD4^+^ subsets, we found that the frequency of T-bet-expressing cells was significantly higher in the CD4^−^CD8^−^ population compared to the CD4^+^ subset (Figure 5C). Additionally, significantly more CD4^−^CD8^−^ iNKT cells were T-bet^hi^ compared to the CD4^+^ population (Figure 5D, gray bars). These findings indicate that CD4^−^CD8^−^ iNKT cells generally express T-bet, while CD4^+^ iNKT cells express T-bet at lower, more variable levels, suggesting that T-bet plays a particularly important role in the function of CD4^−^CD8^−^ iNKT cells.

Eomesodermin was detectable in ~40% of γδ T-cells. In contrast to previous murine studies that were unable to detect Eomes mRNA (21), we found that ~30% of human iNKT cells expressed Eomes protein, although there was significant variation between subjects (Figure 5E). CD4^−^CD8^−^ iNKT cells account for the majority of Eomes expression within the total iNKT-cell population, as a significantly higher frequency of CD4^−^CD8^−^ cells expressed Eomes compared to CD4^+^ iNKT cells (Figure 5E). Despite differences in the frequency of Eomes^+^ cells between iNKT subsets, both subgroups expressed similar levels of Eomes on a per cell basis (Figure 5F). Taken together, these data indicate that Eomes is differentially expressed in human iNKT cells compared to murine iNKT cells and suggest a role for Eomes in the context of human iNKT cells.

In both total γδ T-cells and total iNKT cells, the majority of Eomes^+^ cells co-expressed T-bet, whereas Eomes^−^ cells were either T-bet^lo^ or T-bet^−^ (Figure 5G). Greater than 60% of CD4^+^ iNKT cells did not express T-bet or Eomes, and the remainder were T-bet^lo^ Eomes^−^. CD4^−^CD8^−^ iNKT cells contained substantial T-bet^hi^ Eomes^+^ and T-bet^lo^ Eomes^+^ populations, both of which occurred at a significantly higher frequency than in CD4^+^ iNKT cells. These findings indicate that T-bet and Eomes are highly co-expressed in the CD4^−^CD8^−^, but not in CD4^+^ iNKT cells, suggesting T-bet and Eomes could cooperatively function in CD4^−^CD8^−^ NKT cells.

### T-bet and Eomes are highly expressed in human natural killer cells

**Figure 6:**
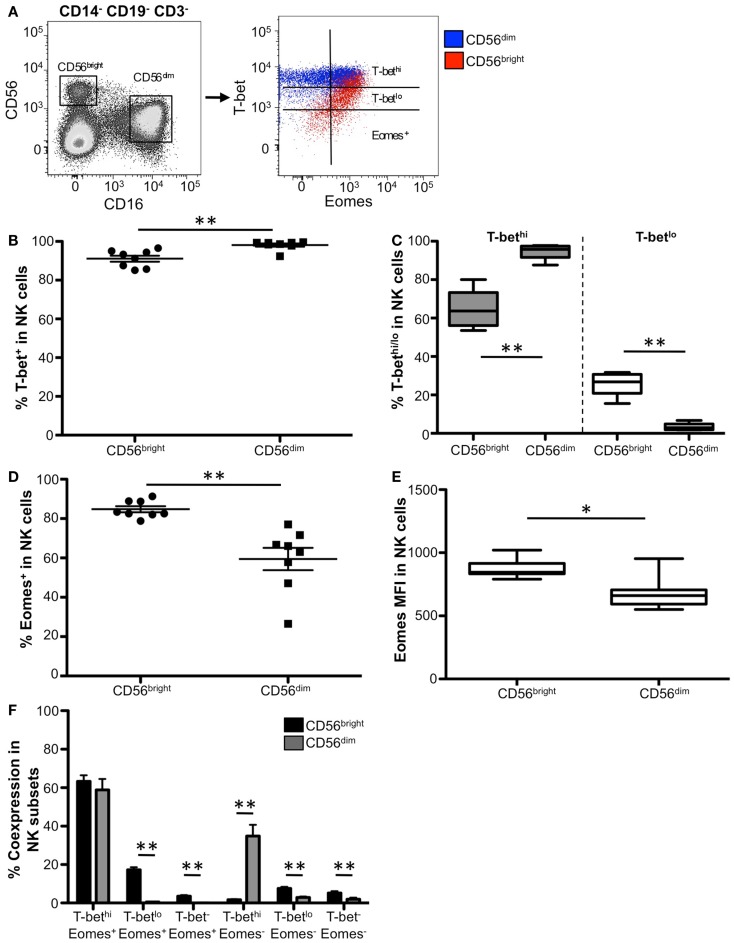
**Distinct T-bet and Eomes expression patterns between CD56^bright^ and CD56^dim^ NK cells**. **(A)** Gating strategy for identifying NK cell subsets. NK subpopulations were gated as follows: singlets, lymphocytes, Aqua Blue^−^ (live cells), and CD14^−^CD19^−^CD3^−^. Mature NK cell populations, CD56^bright^ (CD56^hi^ CD16^−^) and CD56^dim^ (CD56^lo^ CD16^+^), from a representative donor are gated. T-bet and Eomes expression in CD56^bright^ cells (red) and CD56^dim^ cells (blue) are plotted. **(B)** The frequency of T-bet expression in CD56^bright^ and CD56^dim^ NK cells is shown. Each symbol represents and individual donor. **(C)** Box and whisker graphical representation of the frequency of T-bet^hi^ (gray) and T-bet^lo^ (white) expression for each NK population is shown. The box and whisker graphs display 25–75% (box), 10–90% (whisker), and the median value (line). **(D)** The frequency of Eomes-expressing NK cells is shown. **(E)** Eomes MFI from Eomes^+^ cells from each NK population is shown. **(F)** Frequency of T-bet and Eomes co-expression within each subset. *p < 0.04.

In mice, T-bet and Eomes modulate many NK cell effector functions, including cytotoxicity and cytokine production (21, 48). Additionally, their expression is crucial for murine NK developmental regulation where they cooperate to influence several key developmental checkpoints (49). T-bet and Eomes have been highly studied in mouse models, but there are few studies investigating the expression patterns of T-bet and Eomes within human NK cell populations; therefore, we next assessed T-bet and Eomes in human NK subsets. We identified two mature NK cell populations within CD14^−^CD19^−^CD3^−^PBMCs based upon CD56 and CD16 expression (Figure 6A), here referred to as CD56^bright^ (CD56^hi^ CD16^−^) and CD56^dim^ (CD56^lo^ CD16^+^) cells. We observed a gradient of T-bet expression with the CD56^bright^ population (Figure 6A, right panel). While virtually all mature NK cells expressed T-bet, both the frequency of T-bet^+^ cells and the amount of T-bet per cell was significantly greater in the CD56^dim^ population compared to the CD56^bright^ population (Figures 6B,C, gray bars). Conversely, the T-bet^lo^ population was significantly larger in CD56^bright^ cells (white bars). Taken together, these data suggest that there may be an association between T-bet expression levels and functional capacity in NK cells: CD56^dim^ NK cells highly express T-bet and are highly cytotoxic, while poorly cytotoxic CD56^bright^ NK cells express less T-bet and function mainly to produce cytokines (50).

While Eomes was expressed in both CD56^bright^ and CD56^dim^ NK cells, a significantly higher frequency of CD56^bright^ cells were Eomes^+^ compared to CD56^dim^ cells (Figure 6D). Additionally, Eomes^+^ CD56^bright^ cells expressed significantly more Eomes on a per cell basis than Eomes^+^ CD56^dim^ cells (Figure 6E), suggesting that Eomes is likely crucial for CD56^bright^ function.

We next investigated the co-expression of T-bet and Eomes within the CD56^dim^ and CD56^bright^ NK populations. The majority of both NK cell subpopulations were T-bet^hi^ Eomes^+^ (Figure 6F); however, in the remaining cells we found unique co-expression patterns of these transcription factors between the NK subsets. Approximately 15% of CD56^bright^ NK cells were T-bet^lo^ Eomes^+^, and this subpopulation was virtually non-existent in the CD56^dim^ cells. Conversely, ~35% of CD56^dim^ NK cells were T-bet^hi^ Eomes^−^, compared to CD56^bright^ cells which did not have this population. Taken together, these results suggest that while T-bet and Eomes likely play complementary or cooperative roles in the majority of NK cells, there may be distinct subpopulations of NK cells where T-bet and Eomes differentially regulate NK cell function.

### T-bet is predominantly expressed in mature human B-cell plasmablasts

Murine studies have revealed that T-bet is expressed in lymphoid tissue B-cells, where it regulates functional processes such as class switching (51, 52) and homing (53). A recent study of T-bet in B-cells revealed that T-bet is initially expressed during the primary immune response and expression is maintained in IgG2a^+^ memory B-cells, where it is necessary for cell survival and secondary immune responses (54). Additionally, there is evidence to suggest that expression of T-bet in B-cells is necessary for clearance of gHV68, a murine herpes virus (55). While it is appreciated that T-bet is necessary for appropriate B-cell function and antibody responses in mice, T-bet expression is not well-characterized in human B-cells.

**Figure 7:**
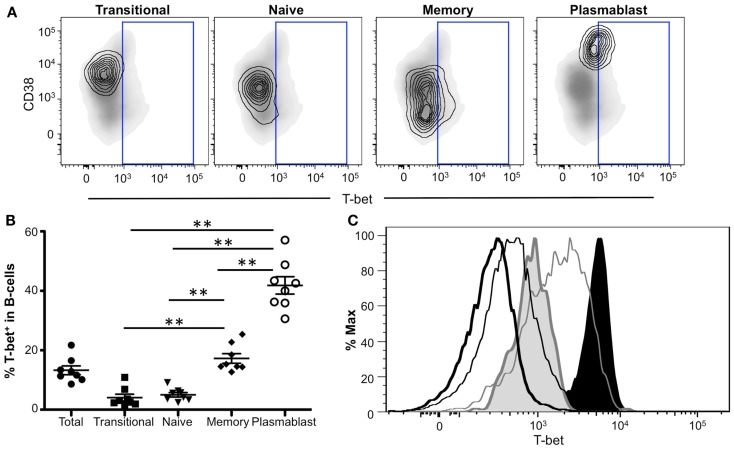
**T-bet expression in antigen-experienced B-cells**. **(A)** T-bet gating strategy for B-cell populations is shown. Transitional, naïve, memory B-cells, and plasmablasts populations are depicted as a contour plot overlaying a density plot of total B-cells. T-bet^+^ events are gated from a representative donor. **(B)** The frequency of T-bet^+^ B-cells within B-cell subpopulations is shown. Each symbol represents an individual subject. Statistical differences of interest, as measured using non-parametric Wilcoxon matched, paired two-tailed *t* tests, are described in the text. *p < 0.04. **(C)** Histograms depicting T-bet expression levels in B-cells and NK cells from a representative donor. Histograms represent the following subsets: naïve B-cells (thick black line), memory B-cells (thin black line), plasmablasts (shaded gray), CD56^bright^ NK cells (gray line), and CD56^dim^ NK cells (shaded black).

To identify B-cell subpopulations, CD19^+^ PBMCs were phenotyped using several B-cell markers: IgD, CD10, CD38, and CD27 (Figure S2 in Supplementary Material). Our analysis focused specifically on transitional/immature B-cells (IgD^+^CD10^+^ CD38^+^CD27^−^), naïve B-cells (IgD^+^CD10^−^CD38^+/−^CD27^−^), memory B-cells (IgD^−^CD10^−^CD38^lo/−^), and plasmablasts (IgD^−^CD10^−^CD38^hi^CD27^+^). Representative flow plots of T-bet expression (contour plot) within each subpopulation are shown in Figure 7A. While Eomes was undetectable in B-cells (data not shown), we found T-bet in ~15% of B-cells (Figure 7B). This T-bet expression was largely relegated to memory B-cells and plasmablasts, with significantly lower amounts observed in transitional/immature and naïve B-cells (Figure 7B). Greater than 40% of plasmablasts expressed T-bet, a significantly higher frequency than that of all other B-cell populations, suggesting that T-bet may play a particularly important role in plasmablast function.

We next compared T-bet expression levels within different B-cell populations to other known T-bet-expressing cell types. Plasmablasts expressed the highest amount of T-bet within the B-cell subsets (Figure 7C, shaded gray), while naïve B-cells displayed the lowest T-bet levels (thick black line). Memory B-cells (thin black line) generally expressed intermediate levels of T-bet; however, a small fraction expressed higher T-bet levels compared to plasmablasts. T-bet expression in T-bet^+^ B-cells was relatively low compared to other T-bet^+^ cells, including CD56^dim^ NK cells (shaded black), representing some of the brightest T-bet-expressing cells, and CD56^bright^ NK cells (thin gray line), which express lower levels of T-bet compared to CD56^dim^ cells. Taken together, these data suggest a key role for T-bet in plasmablasts and memory B-cell subsets and further indicate that T-bet is expressed at a lower level in B-cells compared to other T-bet^+^ lymphocytes.

### Human dendritic cells do not express detectable T-bet or Eomes

Previous studies have shown that several non-lymphocyte populations, including myeloid dendritic cells (mDCs) and plasmacytoid dendritic cells (pDCs), can express T-bet transcript following IFNγ stimulation (56–58); however, it is unclear if human resting cells can also express T-bet. Additionally, Eomes has not been investigated in the context of these populations in humans. To characterize the expression of T-bet and Eomes in resting human dendritic cell populations, we defined mDCs as CD3^−^CD14^−^CD19^−^CD11c^+^ and pDCs as CD3^−^CD14^−^CD19^−^CD123^+^CD11c^−^. Neither T-bet nor Eomes protein was detectable in circulating dendritic cell populations (data not shown), suggesting that T-bet may be upregulated only under specific conditions.

## Discussion

In recent years, many studies have contributed to defining the mechanisms of the transcription factors T-bet and/or Eomes in controlling CD8^+^ and CD4^+^ T-cell functions in mice (25, 59–61); however, studies of T-bet and Eomes in the context of human T-cells, as well as in other cells of the human immune system, have been relatively limited. In this study, we characterized the resting expression patterns of the T-box transcription factors T-bet and Eomes in various resting peripheral blood immune cell populations to provide a basic platform for future studies dissecting their functions within these cell subsets.

Similar to studies in mouse lymphocyte populations, T-bet expression increases as peripheral cells become more differentiated (effector CD8^+^ and CD4^+^ T-cells, CD4^−^CD8^−^ iNKT cells, CD56^dim^ NK cells, and memory and plasmablast B-cells). Overall the same relationship holds true for Eomes expression in these populations, with the exception of B-cells, which lack Eomes, and NK cells where the more differentiated CD56^dim^ cells contain less Eomes than their predecessor CD56^bright^ cells. Taken together, our data suggest an essential role for T-bet and Eomes during peripheral terminal and, in some instances, memory cell differentiation. Additionally, our data would suggest that loss of T-bet or Eomes, depending on cell context, during activation of a number of different cell types would greatly diminish cell differentiation capacity and acquisition of terminal effector functions. In HIV, for example, chronic HIV progressors display significantly lower levels of T-bet and its downstream cytotoxic gene target, perforin, within effector CD8 T-cells compared to elite controller counterparts (9) suggesting that if T-bet levels could be increased in these cells, effector function might be restored.

Because T-bet and Eomes are members of the same family of transcription factors and because they are both associated with effector memory differentiation, they have proposed redundant roles in specific cell types. However, our co-expression analysis reveal that this may not always be the case, and these factors might indeed have unique roles in the context of specific human cell subsets.

Co-expression analysis of T-bet and Eomes indicated that in both CD8^+^ and CD4^+^ T-cells, T-bet^hi^ T_EM_ and effector cells are almost exclusively Eomes^+^ suggesting these two factors cooperate in the context of these subsets. While T-bet^hi^ populations dominate CD8^+^ T_EM_ and effector cells, CD4^+^ T-cells are predominantly T-bet^lo/−^ Eomes^−^, suggesting that in peripheral blood CD4^+^ T-cells T-bet and Eomes likely do not significantly cooperate to modulate CD4^+^ T-cell function. This observation is in contrast to what has been shown previously in mouse studies, although the majority of these studies investigated T-bet and Eomes in the context of splenic CD4^+^ cells (62–65). As T-bet has been shown to control trafficking of lymphocytes through the regulation of chemokine receptor expression (31, 66) it is possible that T-bet^+^ Eomes^+^ CD4^+^ T-cells do not remain in the blood in humans and possibly even in mice, thus explaining the low frequency of co-expressing peripheral CD4^+^ T-cells.

Similar to CD4^+^ and CD8^+^ αβ T-cells, we found that T-bet and Eomes are expressed in a significant subset of the human γδ T-cell population. Previous studies have also observed T-bet and Eomes within mouse γδ T-cells and have linked T-bet and Eomes expression in these cells to IFNγ production (45, 46). Here, we also show that human γδ T-cells co-express T-bet and Eomes and, taken together with mouse studies, these findings suggest that T-bet and Eomes likely also contribute toward IFNγ production and other functions in human cells. Most mouse γδ T-cells constitutively express Eomes and can express T-bet upon stimulation (45, 46), and while a substantial population of human γδ T-cells co-express these factors, a considerable proportion (~50%) of γδ T-cells are T-bet^lo/−^ Eomes^−^. This subset may represent naïve γδ T-cells, which in mice do not express T-bet protein (45), or also may include subsets of γδ T-cells that do not produce IFNγ, such as the IL-17-producing cells in human peripheral blood (67).

Co-expression analysis in NK cell subsets would suggest that as immature NK cells mature into CD56^bright^ cells, Eomes is upregulated and these cells shift from T-bet^lo^ Eomes^+^ to T-bet^hi^ Eomes^+^ CD56^bright^ NK cells. Following appropriate stimuli, CD56^bright^ cells develop into T-bet^hi^ Eomes^+^ CD56^dim^ cells and may gradually lose Eomes expression. Murine studies of transcriptional control support this NK cell maturation model, as Eomes is necessary for the generation and maintenance of mature NK cells (49) and T-bet is necessary to attain the most terminal stages of maturation (21, 49). Alternatively, at least a portion of the T-bet^hi^ Eomes^−^ CD56^dim^ cells may represent developmentally distinct, liver-derived NK cells, which recently have been described in mice as Eomes-independent (68). This subset has not been clearly defined in humans; therefore, further studies are necessary to determine the nature and origin of the T-bet^+^Eomes^−^ NK cell population we have identified in human peripheral blood.

While T-bet and Eomes are best known for their role in cytotoxicity and IFNγ production in T-cells and NK cells, mouse studies indicate that T-bet is also important for the regulation of B-cell antibody class switching (51, 52) and maintenance of IgG2a^+^ memory B-cells (54). We found that T-bet is not significantly expressed in transitional/immature and naïve B-cells, but is detectable in a subset of memory B-cells and is highly expressed in plasmablasts. These data support a model where T-bet likely is not required during early peripheral B-cell development and is first expressed during the germinal center reaction, where it regulates class switching. As class-switched B-cells mature, T-bet likely plays a role in regulating other key functions of these cells. For example, T-bet may regulate homing of effector B-cells to sites of inflammation, as CXCR3 expression is controlled by T-bet in mouse B-cells (53). High frequencies of T-bet expression in plasmablasts indicate the importance of T-bet in these cells; however, mechanistic studies will be necessary to better understand the functions of T-bet in post-germinal center B-cells.

In summary, we have described T-bet and Eomes expression in carefully delineated resting human PBMC subsets and have identified novel cell populations that express T-bet and/or Eomes in resting states. Taken together, our findings suggest potential novel functions for T-bet and Eomes in the context of a number of immune cell subsets and lay the foundation for future mechanistic work to define their numerous roles in human immune cells.

## Author Contributions

James J. Knox, Michael R. Betts, and Laura M. McLane designed the study and developed the methodology; James J. Knox and Laura M. McLane performed and analyzed the experiments and wrote the manuscript; Gabriela L. Cosma performed the experiments.

## Conflict of Interest Statement

The authors declare that the research was conducted in the absence of any commercial or financial relationships that could be construed as a potential conflict of interest.

## Supplementary Material

The Supplementary Material for this article can be found online at http://www.frontiersin.org/Journal/10.3389/fimmu.2014.00217/abstract

Figure S1 **T-bet and Eomes expression in CD8^+^ and CD4^+^ T-cell memory populations**. **(A–D)** T-bet and Eomes expression in CD8^+^ T-cells. **(A)** The frequency of CD8^+^ T-cell memory populations within total CD8^+^ T-cells is shown. Populations were defined as described in the text using the memory markers CCR7, CD45RO, and CD27. **(B)** Box and whisker graphs displaying the frequency of T-bet^hi^ (grey) and T-bet^lo^ (white) cells within each CD8^+^ memory subset. The box and whisker graphs display 25–75% (box), 10–90% (whisker), and the median value (line). **(C)** The frequency of Eomes^+^ cells within each CD8^+^ memory subset is shown. **(D)** Eomes MFI in CD8^+^ memory subsets is displayed using box and whisker graphs. **(E–H)** T-bet and Eomes expression in CD4^+^ T-cells. **(E)** The frequency of CD4^+^ T-cell memory populations within total CD4^+^ T-cells is shown. **(B)** Box and whisker graphs displaying the frequency of T-bet^hi^ (grey) and T-bet^lo^ (white) cells within each CD4^+^ memory subset. The box and whisker graphs display 25–75% (box), 10–90% (whisker), and the median value (line). **(C)** The frequency of Eomes^+^ cells within each CD8^+^ memory subset is shown. **(D)** Eomes MFI in CD4^+^ memory subsets is displayed using box and whisker graphs. *p < 0.04, ** *p* < 0.004.

Figure S2 **B-cell subset identification**. Our method for identifying B-cell subpopulations via flow cytometry is depicted. B-cells are selected as CD19^+^ cells within CD3^−^CD14^−^CD16^−^PBMCs. IgD^+^ B-cells are separated by CD10 expression into CD10-naïve B-cells (CD19^+^IgD^+^CD10^−^CD27^−^) and CD10^+^ transitional/immature B-cells (CD19^+^IgD^+^CD10^+^CD27^−^). IgD^−^ B-cells are separated by CD38 expression into CD38^hi^ plasmablasts (CD19^+^IgD^−^CD38^hi^CD27^+^) and CD38^lo/−^ memory B-cells (CD19^+^IgD^−^CD38^lo/−^).
