# Corrigendum: Characterization of T-bet and Eomes in Peripheral Human Immune Cells

**DOI:** 10.3389/fimmu.2016.00337

**Published:** 2016-09-08

**Authors:** James J. Knox, Gabriela L. Cosma, Michael R. Betts, Laura M. McLane

**Affiliations:** ^1^Department of Microbiology, Perelman Institute for Immunology, University of Pennsylvania, Philadelphia, PA, USA; ^2^Department of Immunology, Thomas Jefferson University, Philadelphia, PA, USA

**Keywords:** T-box transcription factors, T-cells, NK cells, B-cells, T-bet

The corrigendum regards data and text for the final figure of the manuscript, Figure 7:

Subsequent analysis of T-bet levels in human lymphocytes comparing different permeabilization procedures (eBioscience FoxP3 transcription factor kit, BD Pharmingen Cytofix/Cytoperm) has revealed variable findings in the level of T-bet expression detected within certain lymphocyte populations. While this does not change our conclusions for the majority of the populations assessed in this study, B cells in particular show differences under these conditions. Specifically, permeabilization *via* the eBioscience FoxP3 transcription factor staining buffer set indicates that subpopulations of memory B cells express significantly higher levels of T-bet (MFI) compared to plasmablasts, and that plasmablasts express T-bet only at low levels. Subsequent RNA transcript analysis confirms that plasmablasts express T-bet RNA at a level comparable to naïve B cells. Together, in combination with fluorescence-minus-one and isotype control studies, these new findings suggest that subsets memory B cells, not plasmablasts, express the highest levels of T-bet in the B cell compartment and plasmablasts express T-bet at a lower frequency than is reported in Figure 7.

Figure 7 Legend should read:

**(C)** Histograms depicting T-bet expression levels in B-cells and NK cells from a representative donor. Histograms represent the following subsets: naïve B-cells (thick black line), memory B-cells (shaded gray), plasmablasts (thin black line), CD56^bright^ NK cells (gray line), and CD56^dim^ NK cells (shaded black).

B-cell results section should be titled “T-bet is predominantly expressed in mature memory B-cells” and should read:

While Eomes was undetectable in B-cells (data not shown), we found T-bet in ~10% of B-cells (Figure 7B). This T-bet expression was largely relegated to memory B-cells, with significantly lower amounts observed in transitional/immature B-cells, naïve B-cells, and plasmablasts (Figure 7B). Greater than 15% of memory B-cells expressed T-bet, a significantly higher frequency than that of all other B-cell populations, suggesting that T-bet may play a particularly important role in memory B-cell function.

The discussion related to T-bet expression in plasmablasts should be reconsidered as follows:

**Figure 7:**
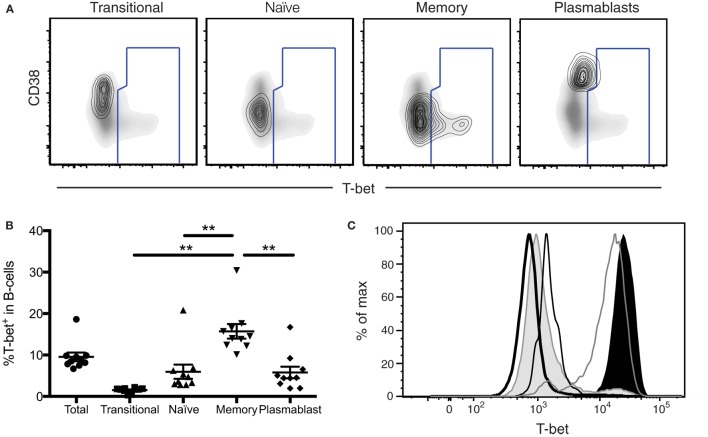
**T-bet expression in antigen-experienced B-cells**. **(A)** T-bet gating strategy for B-cell populations is shown. Transitional, naïve, memory B-cells, and plasmablasts populations are depicted as a contour plot overlaying a density plot of total B-cells. T-bet^+^ events are gated from a representative donor. **(B)** The frequency of T-bet^+^ B-cells within B-cell subpopulations is shown. Each symbol represents an individual subject. Statistical differences of interest, as measured using non-parametric Wilcoxon matched, paired two-tailed *t* tests, are described in the text. *p < 0.04. **(C)** Histograms depicting T-bet expression levels in B-cells and NK cells from a representative donor. Histograms represent the following subsets: naïve B-cells (thick black line), memory B-cells (shaded gray), plasmablasts (thin black line), CD56^bright^ NK cells (gray line), and CD56^dim^ NK cells (shaded black).

We found that T-bet is not significantly expressed in transitional/immature B-cells, naïve B-cells, and plasmablasts, but is highly expressed in subsets of memory-B cells. Reduced frequencies of T-bet expression in plasmablasts indicate a specific role for T-bet at the memory B-cell stage of development, which may no longer be necessary after further differentiation to the plasmablast stage.

## Conflict of Interest Statement

The authors declare that the research was conducted in the absence of any commercial or financial relationships that could be construed as a potential conflict of interest.

