# Supplementary material for: Characterization of T-Bet and Eomes in Peripheral Human Immune Cells
Source: Front Immunol. 2014 May 14;5:217. doi: 10.3389/fimmu.2014.00217 (PMC4030168; doi:10.3389/fimmu.2014.00217)
Supplement: Figure S1 — T-bet and Eomes expression in CD8+ and CD4+ T-cell memory populations. (A–D) T-bet and Eomes expression in CD8+ T-cells. (A) The frequency of CD8+ T-cell memory populations within total CD8+ T-cells is shown. Populations were defined as described in the text using the memory markers CCR7, CD45RO, and CD27. (B) Box and whisker graphs displaying the frequency of T-bethi (grey) and T-betlo (white) cells within each CD8+ memory subset. The box and whisker graphs display 25–75% (box), 10–90% (whisker), and the median value (line). (C) The frequency of Eomes+ cells within each CD8+ memory subset is shown. (D) Eomes MFI in CD8+ memory subsets is displayed using box and whisker graphs. (E–H) T-bet and Eomes expression in CD4+ T-cells. (E) The frequency of CD4+ T-cell memory populations within total CD4+ T-cells is shown. (B) Box and whisker graphs displaying the frequency of T-bethi (grey) and T-betlo (white) cells within each CD4+ memory subset. The box and whisker graphs display 25–75% (box), 10–90% (whisker), and the median value (line). (C) The frequency of Eomes+ cells within each CD8+ memory subset is shown. (D) Eomes MFI in CD4+ memory subsets is displayed using box and whisker graphs. *p < 0.04, ** p < 0.004. [file Presentation1.PPTX]

## Slide 1
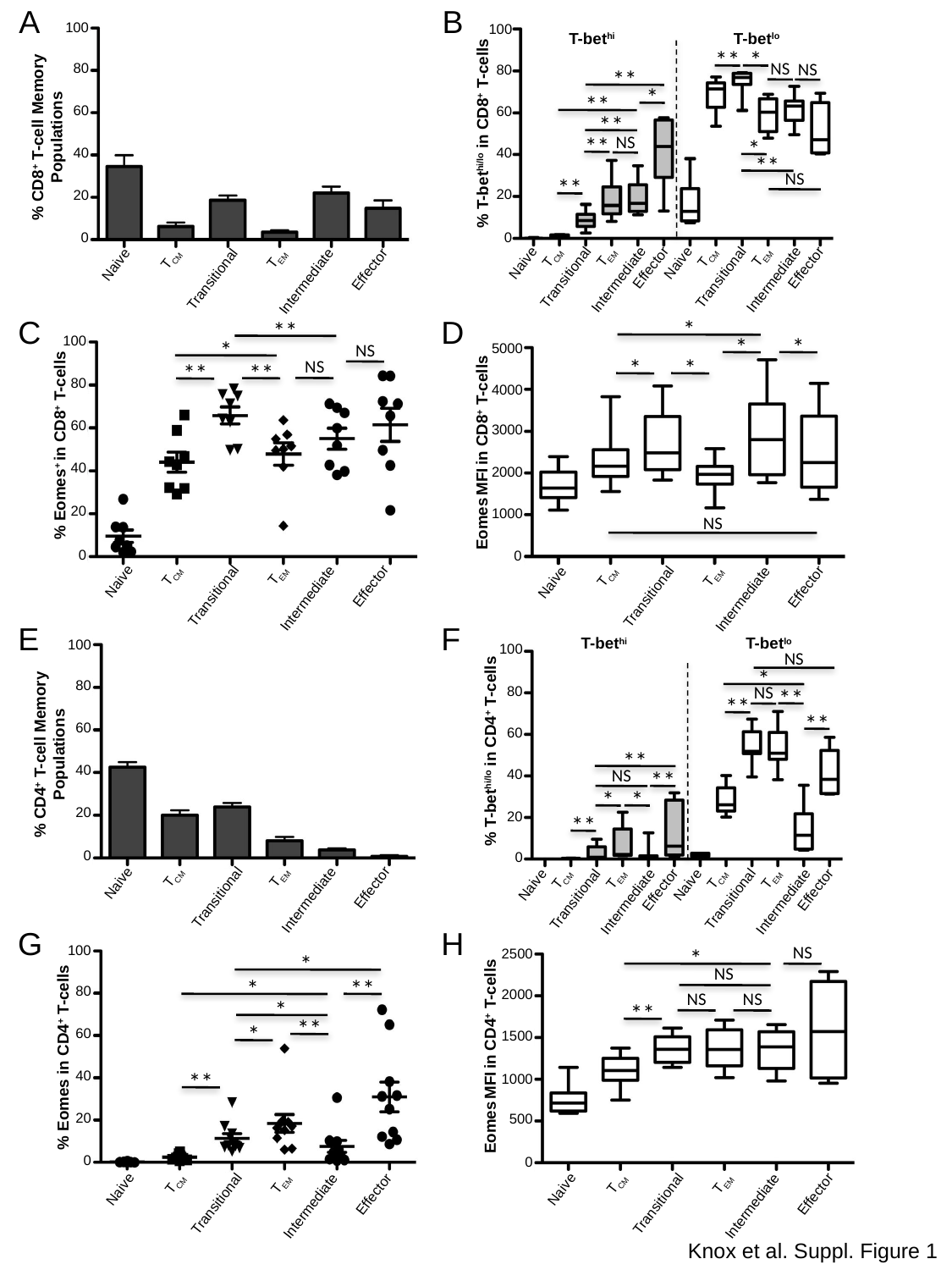

A
B
100
80
60
% CD8+ T-cell Memory Populations
40
20
0
Naive
TCM
Transitional
TEM
Intermediate
Effector
100
T-bethi
T-betlo
**
*
NS
NS
80
**
*
**
60
**
% T-bethi/lo in CD8+ T-cells
NS
**
*
40
**
NS
**
20
0
Naive
TCM
Transitional
TEM
Intermediate
Effector
Naive
TCM
Transitional
TEM
Intermediate
Effector
C
D
*
**
*
*
100
*
NS
5000
4000
3000
Eomes MFI in CD8+ T-cells
2000
1000
0
Naive
TCM
Transitional
TEM
Intermediate
Effector
*
*
NS
**
**
80
60
% Eomes+ in CD8+ T-cells
40
20
NS
0
Naive
TCM
Transitional
TEM
Intermediate
Effector
E
F
T-bethi
T-betlo
100
NS
*
NS
80
**
**
**
60
% T-bethi/lo in CD4+ T-cells
**
NS
40
**
*
*
20
**
0
Naive
TCM
Transitional
TEM
Intermediate
Effector
Naive
TCM
Transitional
TEM
Intermediate
Effector
100
80
60
% CD4+ T-cell Memory Populations
40
20
0
Naive
TCM
Transitional
TEM
Intermediate
Effector
G
H
NS
*
2500
2000
1500
Eomes MFI in CD4+ T-cells
1000
500
0
Naive
TCM
Transitional
TEM
Intermediate
Effector
NS
NS
NS
**
100
80
60
% Eomes in CD4+ T-cells
40
20
0
Naive
TCM
Transitional
TEM
Intermediate
Effector
*
*
**
*
**
*
**
Knox et al. Suppl. Figure 1

## Slide 2
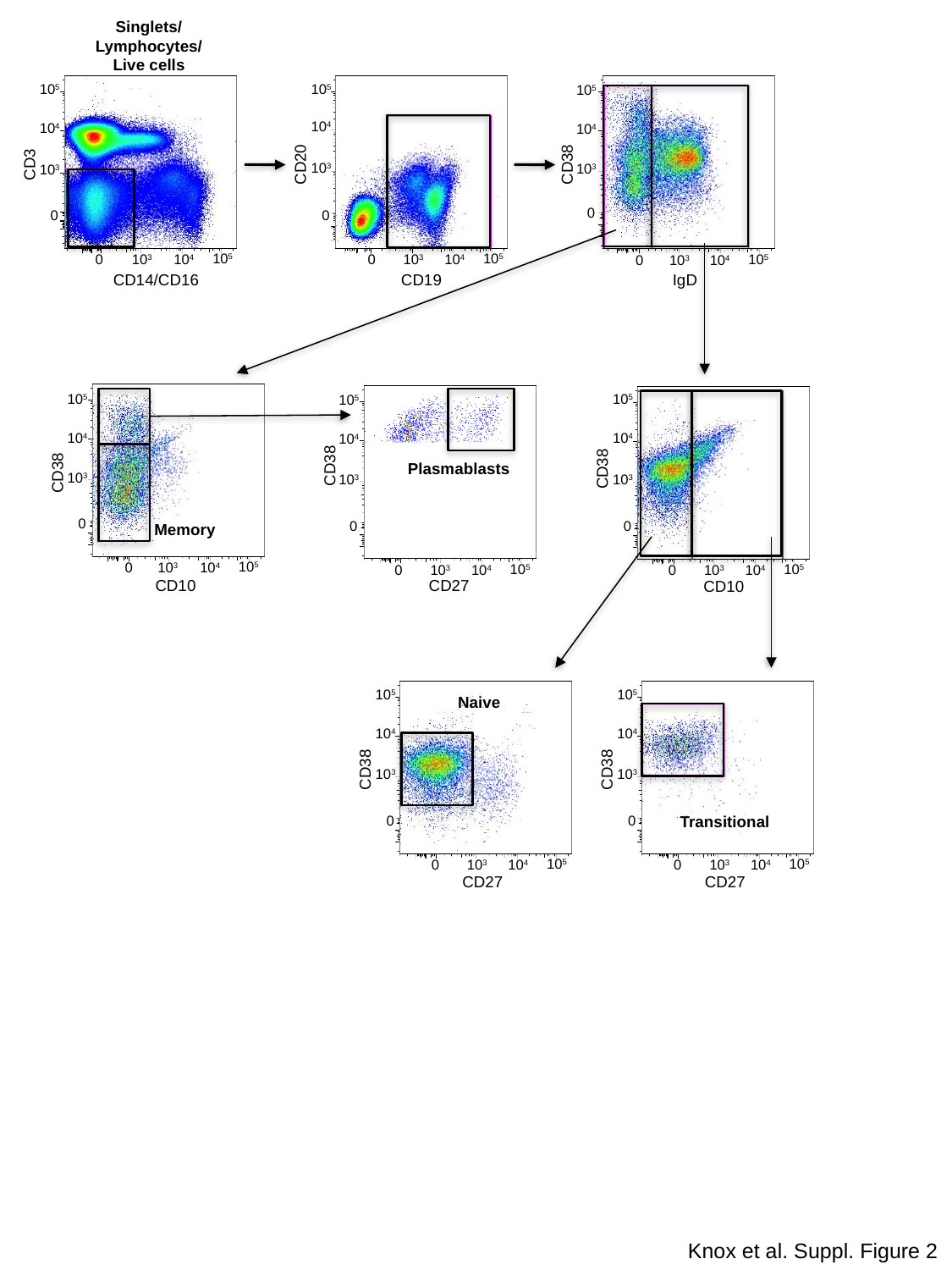

Singlets/Lymphocytes/ Live cells
105
105
105
104
104
104
CD3
CD20
CD38
103
103
103
0
0
0
105
105
0
103
104
0
103
104
105
0
103
104
CD14/CD16
CD19
IgD
105
105
104
CD38
103
0
105
0
103
104
CD10
105
104
104
CD38
Plasmablasts
CD38
103
103
0
0
Memory
105
0
103
104
105
0
103
104
CD10
CD27
105
105
Naive
104
104
CD38
CD38
103
103
Transitional
0
0
105
105
0
103
104
0
103
104
CD27
CD27
Knox et al. Suppl. Figure 2
